# Remimazolam vs propofol for postoperative delirium in adults undergoing general anesthesia: A meta-analysis

**DOI:** 10.17305/bb.2025.12826

**Published:** 2025-08-28

**Authors:** Huijin Zhou, Jing Zhang, Chunyan Du

**Affiliations:** 1 Department of Anesthesiology, Beijing University of Chinese Medicine Third Affiliated Hospital, Beijing, China; 2 Department of Pharmacy, Beijing Huilongguan Hospital, Beijing, China

**Keywords:** Remimazolam, propofol, postoperative delirium, incidence, general anesthesia

## Abstract

Postoperative delirium (POD) is a prevalent and serious complication in adults undergoing surgery with general anesthesia. Remimazolam, an innovative ultra-short-acting benzodiazepine, has been identified as a potential alternative to propofol due to its advantageous pharmacological properties. However, its impact on POD remains uncertain. This study conducted a systematic review and meta-analysis following PRISMA guidelines. A comprehensive search of the PubMed, Embase, Cochrane Library, Web of Science, CNKI, and Wanfang databases was performed up to March 29, 2025. Randomized controlled trials (RCTs) comparing remimazolam and propofol in adult surgical patients under general anesthesia, specifically reporting on POD incidence, were included. A random-effects model was utilized to calculate pooled odds ratios (ORs) with 95% confidence intervals (CIs), accounting for heterogeneity. The analysis included seventeen RCTs encompassing 3133 patients. Overall, remimazolam significantly decreased the risk of POD compared to propofol (OR: 0.71, 95% CI: 0.52–0.97, *P* ═ 0.03; *I*^2^ ═ 36%). Sensitivity analyses, which involved excluding one study at a time, yielded consistent results, reinforcing the robustness of the findings. Subgroup analyses revealed uniform effects across different study designs (single-blind vs double-blind; OR: 0.73 vs 0.64; *P* ═ 0.71) and age groups (adults vs elderly; OR: 0.64 vs 0.72; *P* ═ 0.79). A trend toward greater benefit was observed in studies with longer follow-up periods (7 days: OR: 0.42) and in those employing the CAM or CAM-ICU for POD diagnosis, although subgroup differences were not statistically significant. In conclusion, remimazolam is associated with a significantly reduced risk of POD compared to propofol in adults undergoing general anesthesia.

## Introduction

Postoperative delirium (POD) is an acute and fluctuating disturbance in attention, awareness, and cognition that typically manifests within days following surgery, particularly in elderly or high-risk patients [1, 2]. POD occurs in approximately 10%–50% of adults undergoing major surgery with general anesthesia, with prevalence rates even higher among older individuals and those with comorbidities or preexisting cognitive impairment [3]. The onset of POD is independently linked to multiple adverse outcomes, including extended hospitalization, increased risk of postoperative complications, long-term cognitive decline, and higher mortality rates [4, 5]. Consequently, identifying modifiable perioperative risk factors and implementing preventive strategies is essential for enhancing patient outcomes [6].

Anesthetic agents have garnered increasing attention as significant contributors to POD due to their direct impact on central nervous system function [7, 8]. Recent research aligns with the growing interest in pharmacologic strategies aimed at enhancing postoperative neurocognitive outcomes. Specifically, a study indicated that the administration of parecoxib was linked to improved postoperative cognitive function in elderly patients [9].

Notably, remimazolam—a novel ultra-short-acting benzodiazepine—targets gamma-aminobutyric acid type A (GABA_A) receptors, providing rapid onset and recovery with minimal drug accumulation [10]. Its unique pharmacokinetic profile is characterized by organ-independent metabolism via tissue esterases, stable hemodynamic effects, and a low risk of respiratory depression [11]. These attributes position remimazolam as a promising alternative to propofol for general anesthesia, particularly in vulnerable patient populations [12, 13]. Furthermore, remimazolam may reduce the risk of POD by preventing deep sedation, preserving circadian rhythms, and exerting minimal suppression on cortical arousal and melatonin regulation, although the exact mechanisms underlying these effects require further investigation [14].

Several randomized controlled trials (RCTs) have recently compared remimazolam and propofol for general anesthesia, with some reporting on the incidence of POD [15–31]. While certain trials indicate a lower risk of POD associated with remimazolam [18, 24, 26], the majority of studies reveal comparable outcomes [15–17, 19–23, 25, 27–31]. These findings are influenced by factors such as patient age, type of surgery, anesthetic protocol, and duration of follow-up. Although a few meta-analyses have investigated this issue [14, 32, 33], they are constrained by the limited number of RCTs included. Given the expanding evidence base, the present study seeks to conduct an updated and comprehensive meta-analysis of RCTs to assess the impact of remimazolam compared to propofol on the incidence of POD in adult patients undergoing surgical procedures under general anesthesia.

## Materials and methods

In the design and execution of this study, we adhered to the guidelines established by Preferred Reporting Items for Systematic Reviews and Meta-Analyses (PRISMA) [34, 35] and the Cochrane Handbook [36]. The protocol for the meta-analysis has been registered with International Prospective Register of Systematic Reviews (PROSPERO) under the identifier CRD420251055246.

### Study inclusion and exclusion criteria

This meta-analysis incorporated studies that fulfilled the inclusion criteria outlined by the PICOS framework.

P (Patients): Adult patients (aged 18 years or older) undergoing surgeries under general anesthesia;

I (Intervention): Administration of remimazolam as the primary agent for induction and/or maintenance of general anesthesia;

C (Control): Administration of propofol as the primary agent for induction and/or maintenance of general anesthesia;

O (Outcome): Incidence of POD, with a comprehensive description of diagnostic criteria and assessment tools;

S (Study Design): RCTs with parallel groups.

Studies excluded from the analysis included reviews, editorials, preclinical studies, non-RCT designs, those involving pediatric patients, patients who did not undergo surgery, patients not receiving general anesthesia, studies that failed to compare remimazolam and propofol, those that did not report POD outcomes, or lacked descriptions of diagnostic criteria or assessment tools for POD. In cases where studies involved overlapping patient populations, the study with the largest sample size was selected for inclusion in the meta-analysis.

### Database search

The Medline (PubMed), Embase (Ovid), CENTRAL (Cochrane Library), Web of Science, Wanfang, and CNKI (China National Knowledge Infrastructure) databases were searched using a combination of the following terms: (1) “remimazolam” OR “CNS 7056“ OR “ONO 2745”; (2) “propofol” OR “ICI 35868” OR “disoprofol”; (3) “delirium” OR “confusion” OR “disorientation” OR “cognitive” OR “cognition”; and (4) “random” OR “randomized” OR “randomized” OR “RCT” OR “RCTs” OR “randomly.” Only studies involving human subjects that were published as full-length articles in peer-reviewed journals were included. Grey literature and conference abstracts were excluded due to their lack of peer review and potential deficiencies in methodological detail, which could compromise the reliability and reproducibility of the findings. Additionally, references to relevant reviews and original articles were screened as part of the final database search. This comprehensive search was conducted on March 29, 2025. The detailed search strategy for each database is presented in Supplemental data.

### Data collection and quality evaluation

Two authors conducted independent database searches, data collection, and quality assessments. In cases of disagreement, discussions were held with the corresponding author. A standardized electronic data extraction form was utilized to gather information on study characteristics, patient demographics, interventions, comparators, diagnostic criteria for POD, and outcomes. Inter-reviewer agreement was high, with κ values of 0.88 for data extraction and 0.84 for risk of bias assessment. For studies with overlapping cohorts or duplicate publications, only the dataset with the largest sample size was included.

The collected data encompassed several dimensions, including overall study information (e.g., first author, publication year, and study country), study design (double-blind or single-blind), patient and surgery characteristics (number of patients, mean age, sex, American Society of Anesthesiologists [ASA] class, and type of surgery), details of the intervention involving remimazolam, controls with propofol, follow-up durations, and tools used for diagnosing POD. The quality of the included RCTs was assessed using the Cochrane Risk of Bias Tool [36]. This tool evaluated multiple aspects, including random-sequence generation, allocation concealment, participant blinding, outcome assessment, handling of incomplete outcome data, selective reporting, and other potential sources of bias.

Additionally, two reviewers independently assessed the certainty of evidence utilizing the Grading of Recommendations, Assessment, Development, and Evaluation (GRADE) system, which considers risk of bias, inconsistency, indirectness, imprecision, and publication bias [37]. The certainty of evidence was classified as very low, low, moderate, or high. Disagreements were resolved through discussion with the corresponding author.

**Figure 1.**
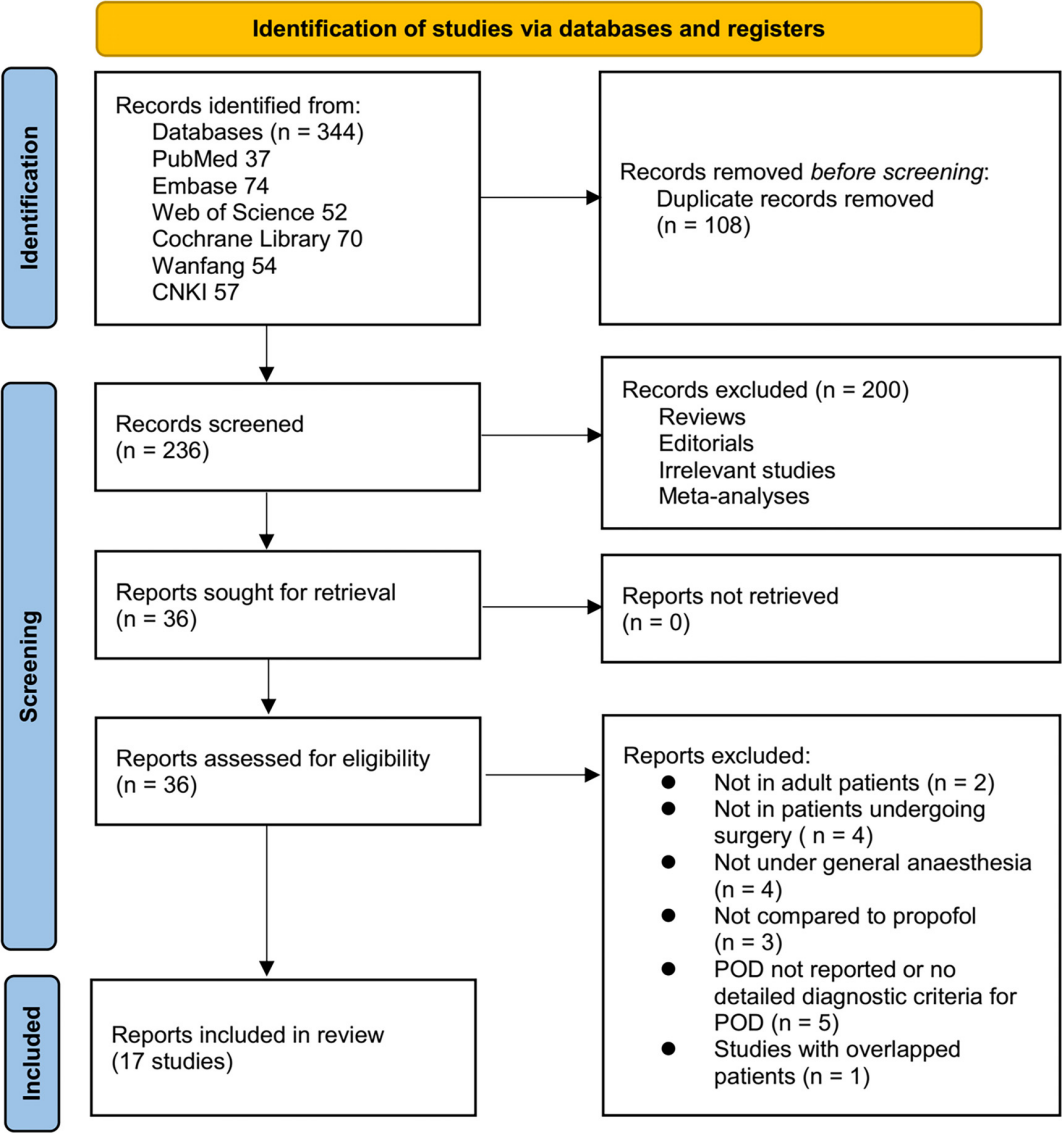
Flowchart for the literature search and study inclusion.

### Statistical analysis

The influence of remimazolam on the incidence of POD was evaluated using odds ratios (ORs) and corresponding 95% confidence intervals (CIs) [36]. Heterogeneity among studies was assessed using the Cochrane *Q* test [36], and the *I*^2^ statistic was calculated, with thresholds of *I*^2^ < 25%, 25%–75%, and >75% indicating mild, moderate, and substantial heterogeneity, respectively [38]. A random-effects model was employed to pool the results, utilizing the generic inverse variance method in RevMan with the DerSimonian–Laird estimator to account for between-study variance, thereby accommodating the potential influence of heterogeneity [36]. For studies with a zero-event arm, we applied a standard continuity correction of 0.5 to both arms, following Cochrane guidelines [36], to facilitate the calculation of OR. Sensitivity analyses were conducted by sequentially excluding one study at a time to assess the robustness of the findings [36]. Additionally, subgroup analyses were performed to investigate the impact of various study characteristics on outcomes, including study design (single-blind vs double-blind), patient age group (adults vs those aged 60 years or older), follow-up durations, and diagnostic tools for POD. The assessment of publication bias was conducted through visual inspection of funnel plots and Egger’s regression asymmetry test [39]. A *P* value of < 0.05 was deemed statistically significant. Statistical analyses were performed using RevMan (version 5.1; Cochrane, Oxford, UK) and Stata software (version 17.0; Stata Corporation, College Station, TX, USA).

**Table 1:** Characteristics of the included randomized controlled trials (RCTs)

**Study**	**Country**	**Design**	**Patients and surgery**	**No. of patients**	**Mean age (years)**	**Men (%)**	**ASA class**	**Intervention (remimazolam)**	**Control (propofol)**	**Follow-up duration (days)**	**Diagnosis of POD**
Mao, 2022	China	R, DB	Adult patients undergoing elective urologic surgery under general anesthesia	128	51.3	67.2	I-III	Induction: 0.2–0.3 mg/kg remimazolam + 0.3–0.5 µg/kg sufentanil; Maintenance: 1–2 mg/kg/h remimazolam + 0.2–0.3 µg/kg/min remifentanil	Induction: 2–3 mg/kg propofol + 0.3–0.5 µg/kg sufentanil; Maintenance: 4–10 mg/kg/h propofol + 0.2–0.3 µg/kg/min remifentanil	1	Nu-DESC
Pan, 2023	China	R, SB	Adult patients undergoing rigid bronchoscopy procedures (tumor resection or stent placement)	30	60.6	90	II-IV	Induction: Remimazolam 0.4 mg/kg IV bolus; Maintenance: Remimazolam 1 mg/kg/h + remifentanil 6–8 µg/kg/h	Induction: Propofol 1.5 mg/kg IV bolus; Maintenance: Propofol 4–8 mg/kg/h + remifentanil 6–8 µg/kg/h	1	Nu-DESC
Yang, 2023	China	R, SB	Adults ≥60 years receiving orthopedic surgery under general anesthesia	300	68.5	39	I-III	Induction: Remimazolam 0.2–0.3 mg/kg + alfentanil 0.04–0.06 mg/kg; Maintenance: Inhaled desflurane (0.3 MAC) + remimazolam (dose titrated to BIS 40–60)	Induction: Propofol 1.0–1.5 mg/kg + alfentanil 0.04–0.06 mg/kg; Maintenance: Inhaled desflurane (0.3 MAC) + propofol (dose titrated to BIS 40–60)	3	CAM
Fechner, 2024	Multiple countries in Europe	R, SB	Adult patients undergoing elective non-cardiac surgery of ≥90 min	365	68	73	III-IV	Remimazolam infusion (mean 1.03 mg/min during surgery), administered from induction to end of surgery; paired with remifentanil	Propofol infusion (mean 4.98 mg/kg/h during surgery), administered similarly with remifentanil	1	Nu-DESC
Li, 2024a	China	R, DB	Adults ≥80 years undergoing elective surgery	146	81	60.8	I-III	Remimazolam: 0.16 mg/kg (ED90) IV bolus over 30s; Rescue dose: 0.05 mg/kg if BIS > 65	Propofol: 0.916 mg/kg (ED90) IV bolus over 30s; Rescue dose: 0.5 mg/kg if BIS > 65	1	CAM
Duan, 2024	China	R, SB	Elderly patients (age 65–90), undergoing hip fracture surgery	106	76.3	46	II-III	Remimazolam: Loading dose: 0.05 mg/kg IV over 1 min; Maintenance: 0.1–0.3 mg/kg/h infusion	Propofol: Loading dose: 0.3–0.5 mg/kg IV over 1 min; Maintenance: 0.5–3 mg/kg/h infusion	7	CAM
Kotani, 2024	Japan	R, SB	Adults ≥20 years undergoing TAVR under general anesthesia	34	83.7	35	NR	Induction: Remimazolam 12 mg/kg/h via IV continuous infusion + Remifentanil (0.2 µg/kg/min); Maintenance: Remimazolam adjusted per SedLine PSI (25–50)	Induction: Propofol 2.5 µg/mL TCI + remifentanil (0.2 µg/kg/min); Maintenance: TCI with adjustments based on SedLine PSI	1	CAM-ICU
Zhang, 2024	China	R, DB	Adults undergoing cerebral endovascular procedures	142	56.3	47.9	I-III	Remimazolam: 0.1 mg/kg IV for induction, 0.3–0.7 mg/kg/h maintenance	Propofol: 1–1.5 mg/kg IV for induction, 4–10 mg/kg/h maintenance	3	CAM-ICU
Liu, 2024a	China	R, SB	Elderly patients (60–80 years), undergoing elective cerebral endovascular surgery under general anesthesia	103	70	46.6	I-III	Remimazolam: Induction: 12 mg/kg/h until loss of consciousness; Maintenance: 1–2 mg/kg/h	Propofol: Induction: 1.5–2 mg/kg; Maintenance: 4–8 mg/kg/h	7	CAM-ICU
Ma, 2024	China	R, SB	Elderly patients (65–80 years) undergoing hip fracture surgery under general anesthesia	80	66.4	40	I-III	Remimazolam: Induction: 0.2–0.4 mg/kg; Maintenance: 0.3–0.5 mg/kg/h	Propofol: Induction: 1.5–2 mg/kg; Maintenance: 4–8 mg/kg/h	3	CAM
Zhou, 2024	China	R, SB	Frail elderly patients (≥60 years) with hip fractures, undergoing hip surgery under general anesthesia	210	67.9	44	NR	Remimazolam: Induction: 0.15–0.35 mg/kg IV bolus; Maintenance: 0.3–1.0 mg/kg/h infusion; Adjunct: Sufentanil 0.4–0.5 µg/kg, cisatracurium 0.2 mg/kg	Propofol: Induction: 1.0–2.5 mg/kg IV bolus; Maintenance: 4–12 mg/kg/h infusion; Same adjunct drugs as intervention	3	3D-CAM
Wang, 2024	China	R, DB	Elderly patients (≥65 years) undergoing lumbar spine surgery	160	72.1	47.5	II-III	Remimazolam: 0.3 mg/kg induction + 0.3–0.8 mg/kg/h maintenance	Propofol: 2.0 mg/kg induction + 4–6 mg/kg/h maintenance	7	CAM
Tian, 2024	China	R, SB	Adults undergoing neurovascular intervention surgery under general anesthesia	98	52	52	I-III	Remimazolam: 0.15 mg/kg/h continuous IV infusion; Adjunct: Remifentanil 0.1–0.3 µg/kg/min	Propofol:2 mg/kg IV bolus; Adjunct: remifentanil 0.1–0.3 µg/kg/min	7	CAM
Liu, 2024b	China	R, DB	Elderly patients (≥65 years) undergoing elective laparoscopic radical resection of colon cancer under general anesthesia	100	71.5	43	I-III	Remimazolam: Induction: 0.1–0.2 mg/kg; Maintenance: 0.4–1.2 mg/kg/h; Adjunct: Sufentanil (0.1–2 µg/kg), cisatracurium (0.2 mg/kg), remifentanil (0.1–0.2 µg/kg/min)	Propofol: Induction: 1–2 mg/kg; Maintenance: 4–10 mg/kg/h; Same adjuncts as intervention group	7	CAM-ICU
Li, 2024b	China	R, SB	Adults (aged 35–59 years) undergoing various laparoscopic surgeries under general anesthesia	84	47.7	52.4	I-II	Remimazolam: Induction: 1–1.5 mg/kg + rocuronium 0.6 mg/kg + sufentanil 0.2 µg/kg; Maintenance: 0.4–0.8 mg/kg/h + rocuronium + remifentanil (0.05–0.2 µg/kg/min)	Propofol: Induction: 1–1.5 mg/kg + rocuronium 0.6 mg/kg + sufentanil 0.2 µg/kg; Maintenance: 4–8 mg/kg/h + rocuronium + remifentanil (0.05–0.2 µg/kg/min)	1	CAM
Fan, 2024	China	R, SB	Elderly patients undergoing cardiac valvular surgery under general anesthesia	319	71.1	42.9	II-III	Remimazolam: Induction: 0.2–0.3 mg/kg; Maintenance: 0.5–1.0 mg/kg/h	Propofol: Induction: 1.0–2.0 mg/kg; Maintenance: 4–10 mg/kg/h	3	CAM-ICU
Fang, 2025	China	R, SB	Older patients (60–90 years) undergoing hip surgery under general anesthesia	728	73	36.1	I-III	Remimazolam: Induction: 0.2–0.25 mg/kg IV; Maintenance: continuous IV infusion (rate not specified); Combined with sufentanil (0.2–0.3 µg/kg), cisatracurium	Induction: 1.5–2.0 mg/kg IV; Maintenance: continuous infusion, titrated to BIS 45–60; Same adjuncts as intervention group	3	3D-CAM or CAM-ICU

Abbreviations: R: Randomized; DB: Double-blind; SB: Single-blind; OL: Open-label; ASA: American Society of Anesthesiologists; NR: Not reported; POD: Postoperative delirium; CAM: Confusion assessmentmethod; CAM-ICU: Confusion assessment method for the intensive care unit; 3D-CAM: 3-minute diagnostic interview for CAM-defined delirium; Nu-DESC: Nursing delirium screening scale; BIS: Bispectral index; IV: Intravenous; TAVR: Transcatheter aortic valve replacement; MAC: Minimum alveolar concentration; PSI: Patient state index; TCI: Target-controlled infusion.

## Results

### Literature search

Figure 1 illustrates the flowchart outlining the database search process and study identification, culminating in the selection of studies for inclusion. Initially, 344 articles were retrieved from the database search, which was subsequently narrowed down to 236 after removing duplicate records. Following this, 200 articles were excluded based on a review of their titles and abstracts, primarily due to their irrelevance to the objectives of the present meta-analysis. Additionally, 19 of the remaining 36 articles were excluded after full-text reviews, for reasons detailed in Figure 1. Ultimately, 17 RCTs [15–31] were identified as suitable for quantitative analysis.

### Study characteristics and data quality

An overview of the included studies is presented in Table 1. This meta-analysis incorporates 17 RCTs [15–31] published between 2022 and 2025. These studies were conducted in China, Japan, and various European countries, collectively enrolling 3133 adult patients undergoing diverse surgeries under general anesthesia. The surgical procedures encompassed orthopedic interventions, urologic surgeries, rigid bronchoscopy, cerebral endovascular procedures, cardiac valve surgery, and neurovascular interventions. The mean age of the participants ranged from 47.7 to 83.7 years, while the proportion of male patients varied between 35.0% and 90.0%.

**Table 2:** Evaluation of study quality using the Cochrane risk-of-bias tool

**Studies**	**Sequence generation**	**Allocation concealment**	**Blinding of participants and personnel**	**Blinding of outcome assessment**	**Incomplete outcome data**	**Selective outcome reporting**	**Other potential threats**
Mao, 2022	Low risk	Low risk	Low risk	Low risk	Low risk	Low risk	Low risk
Pan, 2023	Low risk	Low risk	High risk	Low risk	Low risk	Low risk	Low risk
Yang, 2023	Low risk	Low risk	High risk	Low risk	Low risk	Low risk	Low risk
Fechner, 2024	Low risk	Low risk	High risk	Unclear	Low risk	Low risk	Low risk
Li, 2024a	Low risk	Low risk	Low risk	Low risk	Low risk	Low risk	Low risk
Duan, 2024	Low risk	Unclear	High risk	Low risk	Low risk	Low risk	Low risk
Kotani, 2024	Low risk	Low risk	High risk	Low risk	Low risk	Low risk	Low risk
Zhang, 2024	Low risk	Low risk	Low risk	Low risk	Low risk	Low risk	Low risk
Liu, 2024a	Low risk	Unclear	High risk	Low risk	Low risk	Low risk	Low risk
Ma, 2024	Low risk	Unclear	High risk	Low risk	Low risk	Low risk	Low risk
Zhou, 2024	Low risk	Unclear	High risk	Low risk	Low risk	Low risk	Low risk
Wang, 2024	Low risk	Unclear	Low risk	Low risk	Low risk	Low risk	Low risk
Tian, 2024	Low risk	Unclear	High risk	Low risk	Low risk	Low risk	Low risk
Liu, 2024b	Low risk	Low risk	Low risk	Low risk	Low risk	Low risk	Low risk
Li, 2024b	Low risk	Unclear	Low risk	Unclear	Low risk	Low risk	Low risk
Fan, 2024	Low risk	Unclear	Low risk	Unclear	Low risk	Low risk	Low risk
Fang, 2025	Low risk	Low risk	High risk	Low risk	Low risk	Low risk	Low risk

All included trials compared remimazolam and propofol as anesthetic agents during surgery and reported the incidence of POD. The duration of POD observation spanned 1–7 days, employing various diagnostic tools, such as the Confusion Assessment Method (CAM), the CAM for the Intensive Care Unit (CAM-ICU), the 3-Minute Diagnostic Interview for CAM-defined Delirium (3D-CAM), and the Nursing Delirium Screening Scale (Nu-DESC).

Quality evaluation, utilizing the Cochrane Risk of Bias Tool, indicated that all 17 studies were at low risk of bias regarding random sequence generation and outcome data completeness. Nevertheless, nine studies [15–17, 20, 21, 25, 27, 30, 31] were assessed as having a low risk of allocation concealment. However, blinding of participants and personnel was rated as high risk in 10 studies [16–18, 20–23, 26, 28, 31], reflecting the inherent challenges of maintaining blinding in anesthesia trials. Most studies adequately blinded outcome assessment, although three were classified as unclear in this regard [19, 20, 29]. Overall, there were no indications of selective outcome reporting or other significant threats to validity in any of the studies (Table 2).

### Comparing the influence of remimazolam vs propofol on POD

**Figure 2.**
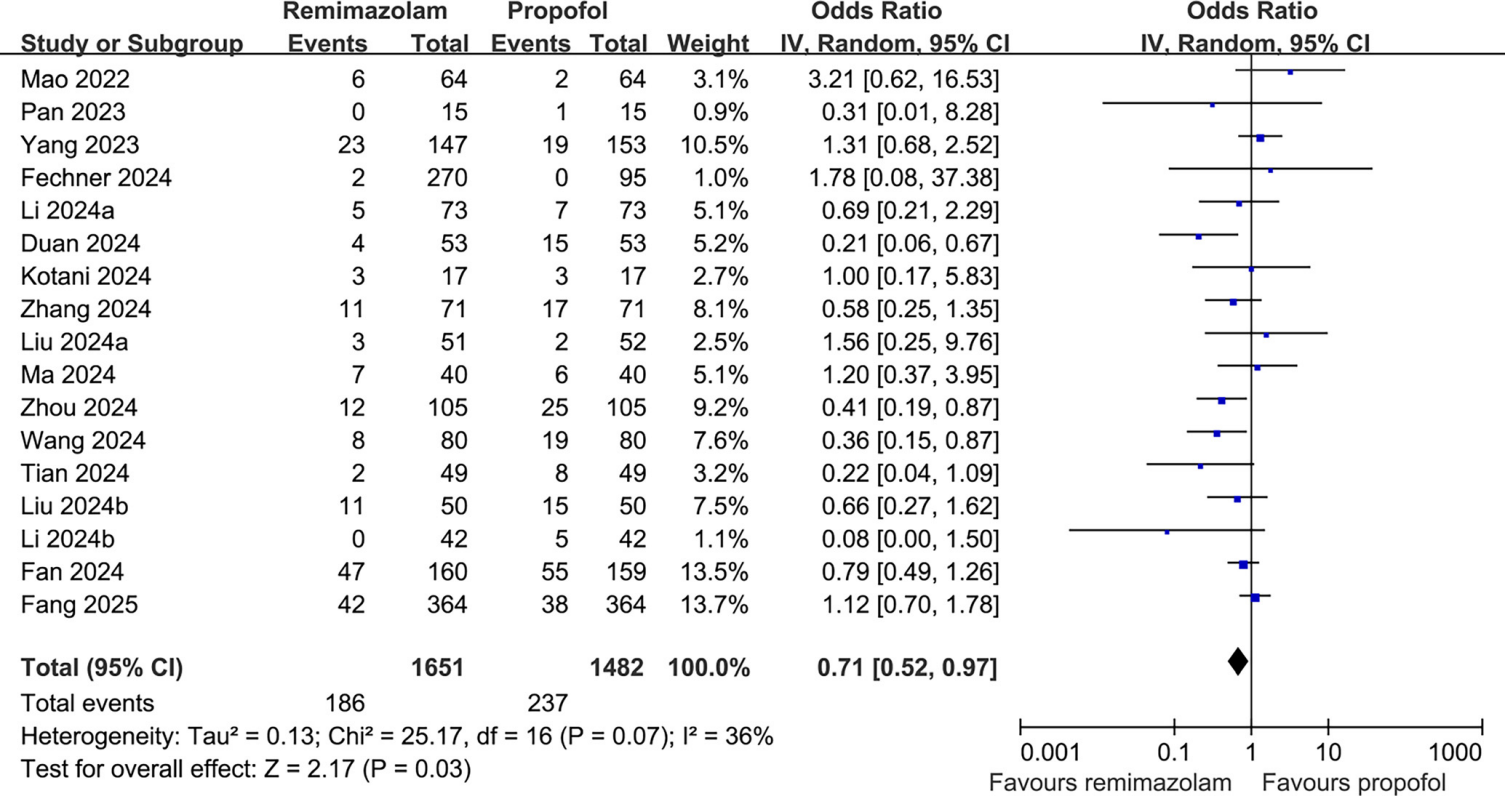
**Forest plot comparing the effect of remimazolam vs propofol on the incidence of postoperative delirium (POD) in adult surgical patients under general anesthesia.** The figure presents individual and pooled ORs with 95% confidence intervals from randomized controlled trials. Abbreviations: POD: Postoperative delirium; OR: Odds ratio; CI: Confidence interval; IV: Inverse variance; RCT: Randomized controlled trial.

A total of 1651 patients were assigned to the intervention group receiving remimazolam, while 1482 patients were allocated to the control group. Among these, 423 patients (13.5%) were diagnosed with POD, including 186 patients (11.3%) from the intervention group and 237 patients (16.0%) from the control group. The pooled analysis of 17 RCTs [15–31] revealed that remimazolam significantly decreased the risk of POD in adult patients undergoing surgeries with general anesthesia compared to propofol (OR: 0.71, 95% CI: 0.52–0.97, *P* ═ 0.03; Figure 2), exhibiting moderate heterogeneity (*I*^2^ ═ 36%). This finding corresponds to an absolute risk reduction of 4.7%, with a number needed to treat of approximately 21, indicating that one case of POD could potentially be prevented for every 21 patients treated with remimazolam instead of propofol.

**Figure 3.**
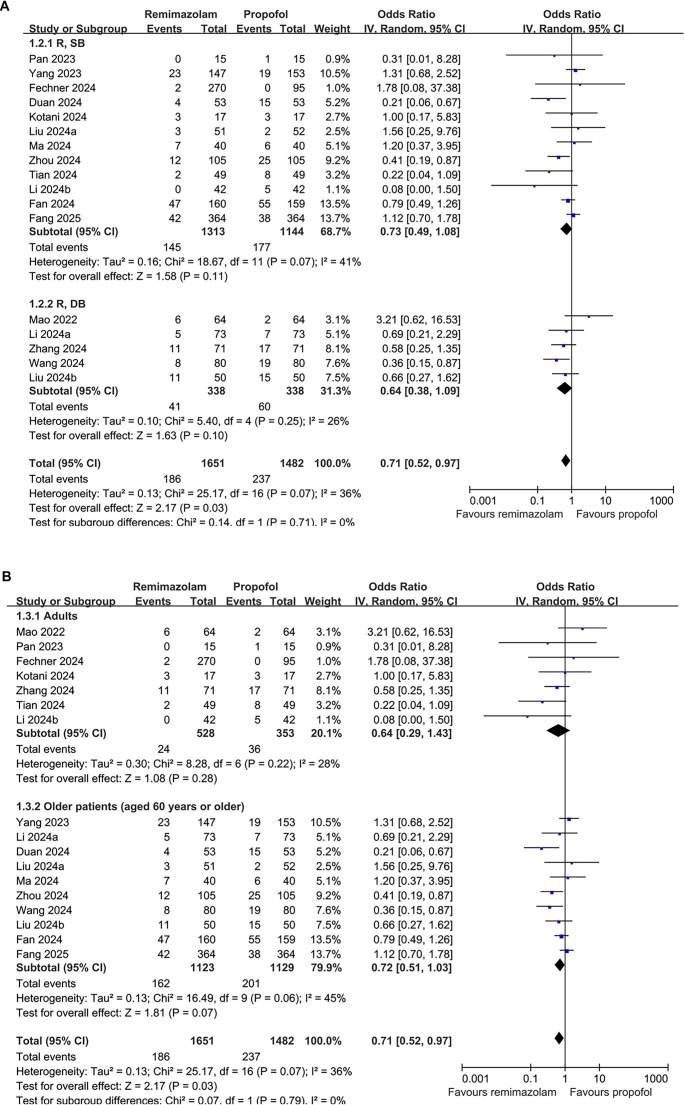
**Forest plot of subgroup analyses comparing the effects of remimazolam versus propofol on the incidence of POD.** (A) Subgroup analysis by blinding method (single-blind vs double-blind); (B) Subgroup analysis by patient age (overall adults vs older patients). Abbreviations: POD: Postoperative delirium; OR: Odds ratio; CI: Confidence interval; R, SB: Randomized single-blind; R, DB: Randomized double-blind.

The summary of evidence certainty, as assessed using the GRADE system, is presented in Table 3. We downgraded the evidence by one level due to potential bias stemming from blinding limitations in certain studies, ultimately categorizing the evidence as moderate certainty. Sensitivity analyses, excluding one study at a time, yielded consistent results (OR: 0.66–0.76, *P* < 0.05 for all). Subsequent subgroup analyses indicated comparable outcomes in single-blind vs double-blind studies (OR: 0.73 vs 0.64, *P* for subgroup difference = 0.71; Figure 3A).

**Table 3:** Summarized certainty of evidence using the GRADE system

**Outcome**	**No. of participants (studies)**	**Design**	**Risk of bias**	**Inconsistency**	**Indirectness**	**Imprecision**	**Other considerations**	**Relative effect (95% CI)**	**Absolute effect**	**Certainty of evidence (GRADE)**	**Comments**
POD incidence (remimazolam vs propofol)	3133 (17 RCTs)	Randomized controlled trials	Serious Some studies had high or unclear risk in blinding	Not serious Moderate heterogeneity (*I*^2^ ═ 36%) but consistent direction of effect	Not serious Population, interventions, and outcomes directly relevant	Not serious 95% CI excludes no effect and is clinically meaningful	None	OR: 0.71 (95% CI: 0.52–0.97)	Risk with propofol: 160 per 1000 risk with remimazolam: 116 per 1000 (95% CI: 88 to 153)	⊕ ⊕ ⊕ ◯ Moderate	Remimazolam reduces POD risk compared with propofol. Findings are based on direct comparisons in surgery patients under general anesthesia. Downgraded for risk of bias due to blinding limitations in some included studies.

Note: Specific reasons for each GRADE domain, including - Risk of bias: Downgraded if a significant proportion of studies had unclear or high risk of bias in key domains (e.g., random sequence generation, 2 > 50%) and could not be explained by subgroup analyses or meta-regression; allocation concealment, or selective reporting); Inconsistency: Downgraded if substantial heterogeneity was observed (I Indirectness: Evaluated but not downgraded, as all included studies directly assessed the population and outcomes of interest; Imprecision: Downgraded if confidence intervals were wide, overlapping no effect, or if the overall sample size was small; Publication bias: Assessed using funnel plots and Egger's test: Downgraded if significant asymmetry suggested potential bias. Abbreviations: GRADE: Grading of Recommendations, Assessment, Development and Evaluation; RCTs: Randomized controlled trials; OR: Odds ratio; CI: Confidence interval.

**Figure 4.**
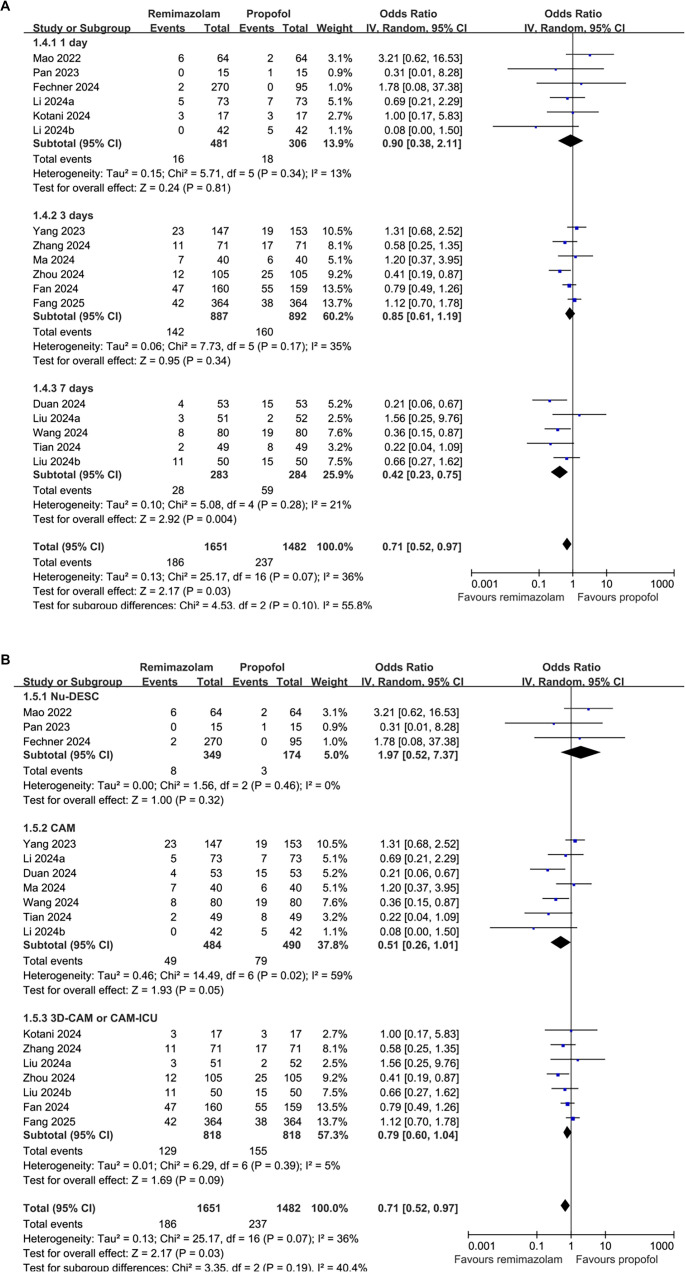
**Forest plot of subgroup analyses comparing the effects of remimazolam vs propofol on the incidence of POD.** (A) Subgroup analysis by follow-up duration (1 day, 3 days, or 7 days); (B) Subgroup analysis by delirium assessment tool (Nu-DESC, CAM, or 3D-CAM/CAM-ICU). Abbreviations: POD: Postoperative delirium; OR: Odds ratio; CI: Confidence interval; Nu-DESC: Nursing delirium screening scale; CAM: Confusion assessment method; CAM-ICU: Confusion assessment method for the intensive care unit.

When focusing exclusively on the five double-blind studies with adequate blinding of both participants and outcome assessment, the association between remimazolam and reduced POD risk remained in the same direction but was no longer statistically significant (OR: 0.64, 95% CI: 0.38–1.09, *P* ═ 0.10; Figure 3A), likely due to diminished statistical power resulting from the smaller sample size. No significant differences were observed between studies involving the overall adult patients and those focusing solely on older patients (OR: 0.64 vs 0.72, *P* for subgroup difference = 0.79; Figure 3B). Notably, remimazolam appeared to be associated with a lower risk of POD in studies with a follow-up duration of 7 days compared to those with follow-up durations of 3 or 1 day (OR: 0.42 vs 0.85 and 0.90), although this difference was not statistically significant (*P* ═ 0.10; Figure 4A). Additionally, remimazolam was linked to a lower risk of POD in studies utilizing the CAM and 3D-CAM/CAM-ICU, but not in studies employing the Nu-DESC (OR: 0.51 and 0.79 vs 1.97). However, the difference between these subgroups was also not statistically significant (*P* ═ 0.19; Figure 4B).

### Publication bias

**Figure 5.**
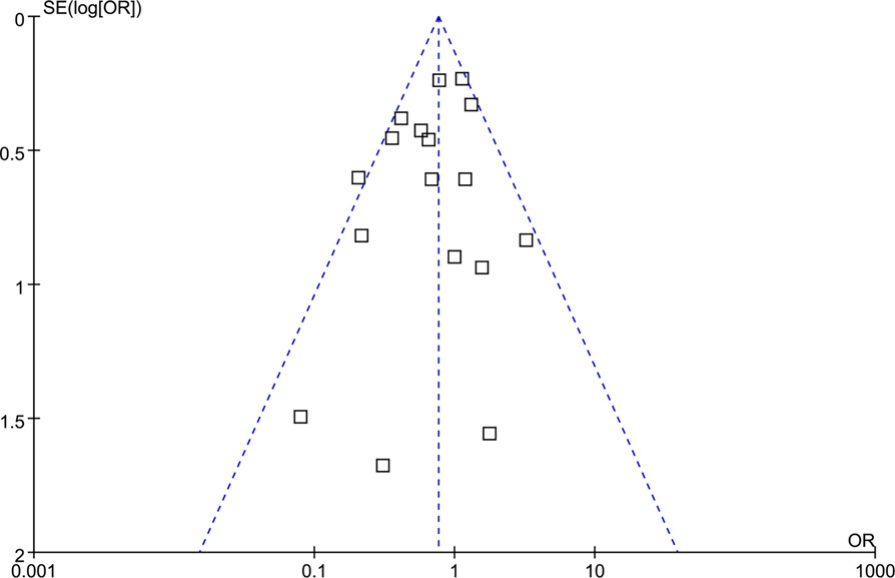
**Funnel plots evaluating the publication bias underlying the meta-analysis comparing remimazolam with propofol on POD.** Visual inspection of the plots reveals symmetry, suggesting a low risk of publication bias. Abbreviations: POD: Postoperative delirium; OR: Odds ratio; SE: Standard error.

The funnel plots for the meta-analysis comparing the effects of remimazolam and propofol on POD are presented in Figure 5. Visual inspection of these plots reveals symmetry, suggesting a low risk of publication bias. Additionally, Egger’s regression test corroborated this finding, indicating a low risk of publication bias (*P* ═ 0.74).

## Discussion

This comprehensive meta-analysis of 17 RCTs involving 3133 adult patients indicates that remimazolam is associated with a significantly reduced risk of POD compared to propofol in surgeries conducted under general anesthesia. Sensitivity analyses confirmed the robustness of these findings, while subgroup analyses demonstrated consistent effects across various study designs (single-blind vs double-blind), age demographics (adults vs elderly), and diagnostic instruments for POD. Although not statistically significant, a trend suggesting greater benefit was identified in studies with extended follow-up periods and those employing validated diagnostic tools like CAM or the CAM for CAM-ICU.

The positive effects of remimazolam in mitigating POD can be attributed to its unique pharmacological and molecular properties. Remimazolam is an innovative, ultra-short-acting benzodiazepine that functions as a GABA_A receptor agonist, akin to midazolam [40]. However, unlike conventional benzodiazepines, remimazolam is rapidly metabolized by tissue esterases into an inactive metabolite, facilitating a swift onset and short duration of action while minimizing drug accumulation, even with extended use [11, 41]. This characteristic contributes to its smooth induction and rapid recovery profile, reducing the risk of oversedation or delayed emergence from anesthesia [42].

In contrast, although propofol is widely utilized for its rapid induction and recovery, it can lead to significant cardiovascular side effects, including hypotension and bradycardia, particularly in elderly or hemodynamically unstable patients [43]. Propofol is also known to induce deep sedation and disrupt the natural sleep–wake cycle, which may interfere with circadian regulation [44]. At the molecular level, propofol interacts not only with GABA_A receptors but also with muscarinic acetylcholine receptors, whose dysfunction is linked to the onset of delirium [45]. Furthermore, propofol is associated with diminished melatonin secretion and disruption of the sleep–wake rhythm, both of which are crucial for cognitive stability postoperatively [46, 47].

In contrast, remimazolam is believed to preserve sleep architecture and maintain more physiological arousal patterns [48]. Its gentle modulation of cortical activity and avoidance of deep sedation may aid in sustaining neural network integrity and cognitive function [49, 50]. These attributes, along with its favorable hemodynamic profile, may enhance its protective effect against POD, particularly in vulnerable populations such as the elderly and individuals with preexisting cognitive risk factors [51].

Our subgroup analyses reinforce the robustness of the overall findings. The effect of remimazolam in reducing POD was consistent across both single- and double-blind studies, as well as among adult and elderly populations. Notably, studies with follow-up durations of seven days or longer demonstrated a stronger protective association, indicating that the benefits of remimazolam may be more pronounced when POD is assessed beyond the early postoperative period. Furthermore, studies using CAM or the CAM-ICU—tools recognized for their high specificity in detecting POD—exhibited a protective association. In contrast, the small Nu-DESC subgroup yielded a point estimate above one (ORs 1.97) with a wide CI, suggesting potential harm but with significant imprecision. This divergence may be attributed to the measurement characteristics, as Nu-DESC is a brief nursing screening tool that is generally less specific than CAM-based instruments. Additionally, differences in case mix or surgical context within the Nu-DESC trials may have influenced the results [52].

Compared to previous meta-analyses, the current study presents several advantages. First, it encompasses a greater number of RCTs and a more diverse patient population. Second, stringent inclusion criteria were implemented, concentrating exclusively on adult patients undergoing general anesthesia and utilizing validated diagnostic tools for POD. Third, multiple sensitivity and subgroup analyses were performed to evaluate the consistency of findings across various study characteristics. In contrast, earlier meta-analyses included fewer studies (6–11) [14, 32, 33], some of which involved mixed anesthesia types or procedural sedation [14, 32], and reported non-significant associations between remimazolam and POD [14, 32, 33]. This meta-analysis, by exclusively examining the intraoperative use of remimazolam during general anesthesia, effectively addresses these limitations and offers a more precise estimate of the treatment effect.

Several limitations must be acknowledged. Moderate heterogeneity was observed (*I*^2^ ═ 36%), likely due to variations in surgical types, patient characteristics, and dosing regimens of remimazolam and propofol. Although most studies focused on elderly or high-risk patients, demographic and clinical variability may impact the risk of POD. Furthermore, the diagnostic criteria and follow-up durations for POD varied among studies, ranging from 1 to 7 days postoperatively, which may lead to underestimation or misclassification of POD incidence. The majority of studies were conducted in China, potentially limiting the generalizability of findings to wider international populations. The predominance of Asian participants, particularly Chinese patients, raises concerns about potential ethnic variations in pharmacogenetics and differences in perioperative care protocols and anesthetic practice patterns, which may influence both baseline POD risk and the comparative effects of remimazolam and propofol. Caution is warranted when extrapolating these findings to non-Asian populations.

Another consideration is the variation in induction and maintenance doses of remimazolam and propofol across the included trials. Such differences may affect the depth of sedation and, consequently, the risk of POD. However, inconsistent reporting and the absence of complete dose data in several studies hindered the calculation of pooled mean doses. This heterogeneity in dosing should be considered when interpreting the results and applying them in clinical practice. Lastly, as this meta-analysis is based on study-level rather than individual patient-level data, residual confounding factors could not be fully addressed.

Clinically, these findings suggest that remimazolam may serve as a safer anesthetic alternative to propofol for patients at risk of POD, particularly among the elderly and those undergoing high-risk surgeries. Its favorable hemodynamic profile and reduced neurocognitive side effects could facilitate recovery and decrease postoperative complications [53]. Future research should explore dose-response relationships, the effects of remimazolam in specific surgical populations (e.g., cardiac and neurosurgical patients), and its comparative efficacy against other anesthetics, such as dexmedetomidine [53]. Additionally, large-scale, multicenter RCTs across diverse healthcare settings are essential to validate these findings.

## Conclusion

In conclusion, this meta-analysis indicates that remimazolam is associated with a significantly lower incidence of POD compared to propofol in adult patients undergoing general anesthesia. Given its pharmacological advantages and consistent efficacy demonstrated across studies, remimazolam may serve as a promising strategy for preventing delirium in perioperative care.

## Supplemental data

**Detailed search strategy for each database**

**PubMed**

(“Remimazolam”[Supplementary Concept] OR remimazolam[tiab] OR “CNS 7056”[tiab] OR “ONO 2745”[tiab]) AND (“Propofol”[MeSH Terms] OR propofol[tiab] OR “ICI 35868”[tiab] OR disoprofol[tiab]) AND (“Delirium”[MeSH Terms] OR delirium[tiab] OR confusion[tiab] OR disorientation[tiab] OR cognitive[tiab] OR cognition[tiab]) AND (“Randomized Controlled Trial”[Publication Type] OR randomized[tiab] OR randomly[tiab] OR RCT[tiab] OR RCTs[tiab])

**Embase**

(‘remimazolam’/exp OR remimazolam:ti,ab OR ‘CNS 7056’:ti,ab OR ‘ONO 2745’:ti,ab) AND (‘propofol’/exp OR propofol:ti,ab OR ‘ICI 35868’:ti,ab OR disoprofol:ti,ab) AND (‘delirium’/exp OR delirium:ti,ab OR confusion:ti,ab OR disorientation:ti,ab OR cognitive:ti,ab OR cognition:ti,ab) AND (‘randomized controlled trial’/exp OR random*:ti,ab OR RCT:ti,ab OR RCTs:ti,ab) AND [humans]/lim

**Cochrane Library (CENTRAL)**

(remimazolam OR “CNS 7056” OR “ONO 2745”) AND (propofol OR “ICI 35868” OR disoprofol) AND (delirium OR confusion OR disorientation OR cognitive OR cognition) AND (randomized controlled trial OR randomized OR randomly OR RCT OR RCTs)

**Web of Science**

TS═(remimazolam OR “CNS 7056” OR “ONO 2745”) AND TS═(propofol OR “ICI 35868” OR disoprofol) AND TS═(delirium OR confusion OR disorientation OR cognitive OR cognition) AND TS═(randomized OR randomly OR RCT OR RCTs)

**CNKI**

(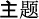: “
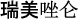
” OR “CNS 7056” OR “ONO 2745”) AND (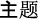: “
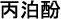
” OR “ICI 35868” OR “disoprofol”) AND (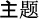: “
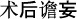
” OR “
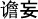
” OR “
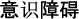
” OR “
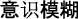
” OR “
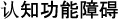
” OR “
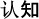
”) AND (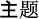: “
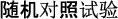
” OR “RCT” OR “
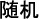
” OR “
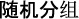
”)

English translation

(Subject: “Remimazolam” OR “CNS 7056” OR “ONO 2745”) AND (Subject: “Propofol” OR “ICI 35868” OR “disoprofol”) AND (Subject: “Postoperative delirium” OR “Delirium” OR “Disorders of consciousness” OR “Disorientation” OR “Cognitive impairment” OR “Cognition”) AND (Subject: “Randomized controlled trial” OR “RCT” OR “Randomized” OR “Random allocation”)

**Wanfang Data**


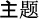
: (“
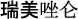
” OR “CNS 7056” OR “ONO 2745”) AND 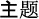: (“
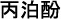
” OR “ICI 35868” OR “disoprofol”) AND 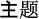: (“
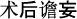
” OR “
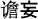
” OR “
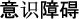
” OR “
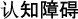
” OR “
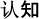
”) AND 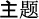: (“
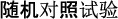
” OR “
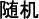
” OR “RCT”)

English translation

Subject: (“Remimazolam” OR “CNS 7056” OR “ONO 2745”) AND Subject: (“Propofol” OR “ICI 35868” OR “disoprofol”) AND Subject: (“Postoperative delirium” OR “Delirium” OR “Disorders of consciousness” OR “Cognitive impairment” OR “Cognition”) AND Subject: (“Randomized controlled trial” OR “Randomized” OR “RCT”)

## Data Availability

All data generated or analyzed during this study are included in this published article.
